# Correction to “Hydrogen Bond–Driven N‑Terminal Amyloid β Mimetics Attenuate Neurotoxic Aggregation”

**DOI:** 10.1021/acs.chemmater.5c02881

**Published:** 2025-11-10

**Authors:** Jia Kong, Jiaxing Zhang, Yirui Zhang, Huanxin Xue, Xin Guo, Nan Wang, Ziyi Gao, Menghuan Sun, Yinqiang Xia, Yuefei Wang, Hao Ren, Peng Yang, Man Shing Wong

In the introduction section, the following argument warrants revision for greater accuracy.

“The p3 peptide, a naturally occurring N-terminally truncated variant derived from the nonamyloidogenic pathway, is considered innocuous.^13,14^”

This correction provides a revision for the above statement as follows:

“The p3 peptide, a naturally occurring N-terminally truncated variant derived from the nonamyloidogenic pathway, has often been regarded as innocuous;^13^ however, its potential amyloidogenic toxicity remains debated.^14^”

We regret any potential misunderstanding caused. The conclusions of our study still stand and are unaffected by this revision.

